# CO_2_ refixation is higher in leaves of woody species with high mesophyll and stomatal resistances to CO_2_ diffusion

**DOI:** 10.1093/treephys/tpab016

**Published:** 2021-02-17

**Authors:** Diana Eckert, Helle Juel Martens, Lianhong Gu, Anna Monrad Jensen

**Affiliations:** Department of Forestry and Wood Technology, Linnaeus University, 351 95 Växjö, Sweden; Department of Geosciences and Natural Resource Management, University of Copenhagen, Rolighedsvej 23, Frederiksberg C, 1958 Copenhagen, Denmark; Climate Change Science Institute & Environmental Sciences Division, Oak Ridge National Laboratory, Oak Ridge, TN 37831-6301, USA; Department of Forestry and Wood Technology, Linnaeus University, 351 95 Växjö, Sweden

**Keywords:** boreal trees, CO_2_ refixation, ecophysiology, leaf mass per area, mesophyll resistance/conductance, photosynthesis, stomatal resistance/conductance

## Abstract

The percentage of respiratory and photorespiratory CO_2_ refixed in leaves (*P*_*r*_) represents part of the CO_2_ used in photosynthesis. The importance of *P*_*r*_ as well as differences between species and functional types are still not well investigated. In this study, we examine how *P*_*r*_ differs between six temperate and boreal woody species: *Betula pendula*, *Quercus robur*, *Larix decidua*, *Pinus sylvestris*, *Picea abies* and *Vaccinium vitis-idaea*. The study covers early and late successional species, deciduous broadleaves, deciduous conifers, evergreen conifers and evergreen broadleaves. We investigated whether some species or functional types had higher refixation percentages than others, whether leaf traits could predict higher *P*_*r*_ and whether these traits and their impact on *P*_*r*_ changed during growing seasons. Photosynthesis CO_2_ response (*A/C_i_*)-curves, measured early, mid and late season, were used to estimate and compare *P*_*r*_, mesophyll resistance (*r*_*m*_) and stomatal resistance (*r*_*s*_) to CO_2_ diffusion. Additionally, light images and transmission electron microscope images were used to approximate the fraction of intercellular airspace and cell wall thickness. We found that evergreens, especially late successional species, refixed a significantly higher amount of CO_2_ than the other species throughout the entire growing season. In addition, *r*_*m*_, *r*_*s*_ and leaf mass per area, traits that typically are higher in evergreen species, were also significantly, positively correlated with *P*_*r*_. We suggest that this is due to higher *r*_*m*_ decreasing diffusion of (photo) respiratory CO_2_ out of the leaf. Cell wall thickness had a positive effect on *P*_*r*_ and *r*_*m*_, while the fraction of intercellular airspace had no effect. Both were significantly different between evergreen conifers and other types. Our findings suggest that species with a higher *r*_*m*_ use a greater fraction of mitochondria-derived CO_2_, especially when stomatal conductance is low. This should be taken into account when modeling the overall CO_2_ fertilization effect for terrestrial ecosystems dominated by high *r*_*m*_ species.

## Introduction

When illuminated, the CO_2_ concentration inside plant leaves, around Rubisco, determines photosynthetic activity (Campbell et al. 1988, Terashima et al. 2011). Although the majority of CO_2_ fixed may be derived from the atmosphere (Parkhurst 1994), there is a substantial amount of CO_2_ produced and released inside mesophyll cells through respiration and photorespiration (TL Sage and RF Sage 2009, Tholen et al. 2012, Sage and Khoshravesh 2016, Walker et al. 2016). The degree to which plants utilize this intercellular source of CO_2_, the significance thereof compared with atmospheric CO_2_, and whether any conditions promote the refixation of (photo)respiratory CO_2_, are all questions to consider when modeling the overall photosynthetic capacity of plants. For example, not incorporating mesophyll resistance to CO_2_ diffusion (*r_m_*) results in up to a 75% underestimation of the maximum carboxylation rate of Rubisco (*V*_cmax_) (Sun et al. 2014*a*). Knowing more about the percentage of (photo)respiratory CO_2_ being refixed, *P_r_* might be similarly important for accurate modeling of terrestrial CO_2_ uptake, especially when considering future increased CO_2_ conditions (Sun et al. 2014*b*, Sage and Khoshravesh 2016).

CO_2_ produced through (photo)respiration either diffuses directly from the mitochondria into the chloroplasts due to their close proximity (Sage and Khoshravesh 2016) or leaks to the intercellular air spaces (von Caemmerer 2013, Walker et al. 2016). Little is known about either of these pathways. If respiratory CO_2_ joins the CO_2_ in the intercellular airspaces, the probability of it diffusing into another cell, and being refixed by Rubisco, should be affected by the same conditions that influence CO_2_ concentrations in the intercellular airspaces overall. One major factor could therefore be *r_m_*, which directly influences the movement of CO_2_ inside leaves (Warren 2008). It is generally observed that lower *r_m_* facilitates CO_2_ uptake and correlates with larger concentrations of CO_2_ around Rubisco, increasing photosynthetic rates (Warren 2008). However, as respiratory CO_2_ is not diffusing all the way from the atmosphere, this does not necessarily hold true for refixation percentages. Rather, higher resistance to CO_2_ diffusion should lower the fraction of respiratory CO_2_ that escapes out of the leaf once it has left the cell it derived from. We therefore predict a positive correlation between *r_m_* and the amount of refixation that occurs.

Several studies show that cell wall thickness and the surface area of chloroplasts exposed to intercellular airspaces are among the most important factors influencing *r_m_* (Tomás et al. 2013, Jimei et al. 2017, Veromann-Jürgenson et al. 2017). However, the level of restriction of CO_2_ diffusion due to different anatomical traits varies among species and foliage structure (Tomás et al. 2013). It is likely that plant species with similar phenotypical characteristics, plant functional types (PFTs), will also have similar factors influencing *r_m_* (Chapin III et al. 1996). Plant functional types are often used in vegetation models for climate and land use monitoring (Lavorel et al. 2007), and while photosynthetic biochemical parameters and species-specific capacities for CO_2_ assimilation are described across PFTs for tree species (Wullschleger 1993, Wright et al. 2004, Niinemets et al. 2015), information on *P_r_* and how it is influenced by *r_m_* is still incomplete. It would therefore be useful to provide more information about the fate of respiratory-derived CO_2_ for different PFTs to increase the accuracy of such models. Some major PFTs that have been shown to have very different physiological and morphological traits in the boreal zone are broadleaved, coniferous, deciduous and evergreen trees (Chapin et al. 1996). Significant differences in the leaf investment strategies of these types may be found. For example, boreal evergreen conifer leaves typically have thick cell walls, sunken stomata and resin channels inside the leaves (Grassi and Bagnaresi 2001, Ghimire et al. 2015, Fan et al. 2019). Morphological differences could be one explanation for significant differences in average *P_r_* between the PFTs. Another way of separating plant species into types is sorting them according to pioneer and climax species. We know, for example, that pioneer species tend to have thinner leaves than climax species (Sobrado 2008, Han et al. 2010), as well as a tendency for higher photosynthetic rates per unit of leaf area (Bazzaz 1979).

In addition to anatomical differences between species and PFTs, leaf structure and organelle position may vary during a growing season: some evergreen broad-leaved tree species, such as for example *Quercus glauca*, have higher leaf mass per area (LMA) and net photosynthetic rates late in the growing season (Miyazawa et al. 1998). Furthermore, Miyazawa and Terashima (2001) found that the surface area of chloroplasts facing the intercellular air spaces on a leaf area basis increased within a period of up to 40 days after full leaf expansion. This merits exploring if *P_r_* is influenced by seasonality and foliar maturation. Our hypothesis is that differences in leaf anatomy, whether due to functional type or leaf development stage, will correlate with differences in *P_r_*.

Stomatal resistance to CO_2_ diffusion (*r_s_*) substantially affects CO_2_ concentrations at the photosynthetic sites (Wong et al. 1979). It is the most direct means for plants to prevent cellular water loss; stomatal closure (high *r_s_*) simultaneously slows diffusion of CO_2_ and preserves water by slowing transpiration (Wong et al. 1979, Schulze 1986, Franks and Farquhar 1999). However, as discussed in connection with *r_m_*, mitochondria-derived CO_2_ does not need to diffuse from the leaf exterior. High *r_s_* might therefore trap CO_2_ produced in the mitochondria and thus correlates with higher *P_r_*. Furthermore, *r_s_* has a tendency to increase with increasing LMA when data are pooled from several PFTs and species (Onoda et al. 2017). Comparing *r_s_* and *P_r_* across species and functional types should therefore give sufficient variation in *r_s_* for a correlation with *P_r_* to potentially be revealed.

In this paper, we investigated anatomical and physiological traits that affected leaf-level CO_2_ diffusion. We considered if these traits also influenced *P_r_*, and compared several plant functional types. In addition, we evaluated if and how these traits changed as the foliage developed throughout the growing season. Specifically, we tested whether:

(i) Evergreen and conifer species will have greater *P_r_* compared with deciduous species. We suspect that this might in part be due to greater values of *r_m_* and *r_s_*, therefore;
(ii) The refixation percentage is positively affected by greater rates of *r_m_* and *r_s_*. As these physiological factors might be affected by leaf maturation, and thus during the growing season when measuring takes place, we therefore finally test whether;
(iii) Leaves show greater *P_r_*, *r_m_* and *r_s_* later in the growing season, and whether differences in LMA, cell wall thickness and average area fraction of intercellular airspace have an effect on *P_r_*, *r_m_* and *r_s_*.

## Materials and methods

### Study site and plant material

The plant material was collected in an open, mixed deciduous forest in Växjö, southern Sweden (56°50′26.6″N 14°49′20.6″E). The 30-year means, monthly air temperature and precipitation from May to September, averages around 12.2 °C and 618 mm. The climate is coastal temperate with mild winters. Data were collected in 2017. The average monthly air temperatures and precipitation in May, July and September that year were 11.9 °C and 16.8 mm, 15.3 °C and 39.7 mm and 12.2 °C and 63.1 mm, respectively (Swedish Meteorological and Hydrological Institute 2018).

Current-year folia samples from mature Roth. *Betula pendula*, L. *Quercus robur*, Mill. *Larix decidua*, L. *Pinus sylvestris*, (L.) Karst. *Picea abies* and L. *Vaccinium vitis-idaea* were collected from a south-facing site at 1.3 m (with the exception of *V. vitis-idaea*, where the whole plant was collected). Individuals were sampled at three different time points during the growing season: early (late May), mid (early July) and late season (late August and early September). *Betula pendula* and *Q. robur* are deciduous broadleaved trees, *L. decidua* is a deciduous conifer tree and *P. sylvestris* and *P. abies* are evergreen conifer trees, whereas *V. vitis-idaea* is an evergreen broadleaved shrub. In southern Sweden, *B. pendula*, *Q. robur*, *L. decidua* and *P. sylvestris* are grouped as early to mid-successional species (primary species), and *P. abies* and *V. vitis-idaea* are late successional species (climax species).

Eight to 10 branches per species were cut, brought back to the laboratory and placed in water, after which the stems were recut while submerged, avoiding cavitation. Other studies using detached branches have shown gas exchange measurements to be stable at least 14 h after sampling (Dang et al. 1997, Mitchell et al. 1999). We measured gas exchange no more than 8 h after branch collection. Leaves adjacent to the ones used for the gas exchange measurements were removed and prepared for microscopy (see Microscopy and leaf trait analysis section). This procedure was repeated at each time point of the growing season with the same species and the number of samples collected at the same site.

### Gas exchange measurements

Gas exchange measurements were done using a 6-cm^2^ chamber of the LI-6400 and LI-6800 with red-blue light emitting diodes light sources (LICOR Inc., Lincoln, NE, USA). The reference CO_2_ concentration was set at 400 p.p.m., block temperature at 25 °C, flow rate at 500 μmol s^−1^, relative humidity at 50 ± 10% and irradiance at 1000 μmol quanta m^−2^ s^−1^. These conditions were kept constant while the leaves acclimated to the chamber. The leaves were considered acclimated when the photosynthetic rate and stomatal conductance had reached steady state (usually achieved after 20–40 min). Photosynthesis CO_2_ response (*A/C_i_*) curves were generated by sequential adjustment of the reference CO_2_ concentration between 50 and 1200 p.p.m. (400, 300, 200, 100, 50, 400, 600, 800, 1000 and 1200 p.p.m.). A total of 156 *A/C_i_* curves were generated. Values for stomatal resistance *r_s_* included in the results were taken at the first 400-p.p.m. CO_2_ concentration.

*A/C_i_* curves were analyzed with the LeafWeb online tool and database (www.leafweb.org), which estimated the maximum rate of CO_2_ fixation (*A*_max_), *V*_cmax_, the maximum rate of electron transport for a given light intensity (*J*_max_), maximum rate of triose phosphate use (TPU), *r_m_* and *P_r_* by fitting a modified version of the Farquhar--von Caemmerer--Berry model (Gu et al. 2010). LeafWeb calculates refixation percentage according to the equation presented by Tholen et al. (2012). The equation uses the fraction of respiratory and photorespiratory CO_2_ that has not escaped to the atmosphere; it includes the cytosolic partial pressure of mitochondrial CO_2_, the diffusion resistance to these molecules imposed by the chloroplasts, the resistance represented by carboxylation reaction itself, and the resistance derived by the cell wall and plasma membrane. The estimated amount of (photo)respiratory CO_2_ that escapes to the atmosphere is the leakage flux divided by the sum of the two fluxes originating in the mitochondria (Tholen et al. 2012). Hence, the relative fraction of respiratory and photorespiratory CO_2_ presumed to be refixed and used in photosynthesis, *P_r_*, is estimated as 1 minus the relative amount of CO_2_ that escapes to the atmosphere, calculated according to Tholen et al. (2012)

$$\begin{eqnarray*} {P}_r&=&1-\left(\frac{x_{\mathrm{py}}}{r_{\mathrm{wp}}+{r}_{\mathrm{sc}}}/\left(\frac{x_{\mathrm{py}}}{r_{\mathrm{ch}}+{k}^{-1}}+\frac{x_{\mathrm{py}}}{r_{\mathrm{wp}}+{r}_{\mathrm{sc}}}\right)\right.\nonumber\\ &=&\left.{\left(\frac{r_{\mathrm{wp}}+{r}_{\mathrm{sc}}}{r_{\mathrm{ch}}+{k}^{-1}}+1\right)}^{-1}\right). \end{eqnarray*}$$

Here, *r*_ch_ is the resistance represented by the chloroplast, *x*_py_ is the cytosolic partial pressure of (photo)respiratory CO_2_ molecules, and resistance from the cell wall, plasma membrane and stomata is indicated as *r*_wp_ and *r*_sc_, respectively. In addition, the resistance derived from the carboxylation reaction itself was included (${k}^{-1}\equiv ({p}_c-0)/{V}_c$). For the reported values, the resistances are expressed in Pa s^−1^ m^−2^ μmol^−1^ because diffusion inside the leaf is a process driven by the gradient in partial pressure (e.g., Pa) rather than concentration. The conversion factor between *r_m_* (m^2^ s Pa μmol^−1^) and *r_m_* (m^2^ s mol^−1^) is given by

$$ {r}_m\ \mathrm{in}\ {\mathrm{m}}^2\ \mathrm{s}\ \mathrm{Pa}\ {\mu \mathrm{mol}}^{-1}=\mathrm{P}\times{10}^{-6}\ {r}_m\ \mathrm{in}\ {\mathrm{m}}^2\ \mathrm{s}\ {\mathrm{m}\mathrm{ol}}^{-1}. $$

Here, *P* is the total atmospheric pressure in Pa.

After gas exchange measurements, projected leaf area for *L. decidua*, *P. sylvestris*, *P. abies* and *V. vitis-idaea* was calculated using ImageJ (Schneider et al. 2012) and adjusted in the gas exchange output accordingly. All samples (either a 2.27-cm^2^ leaf disc from *B. pendula* and *Q. robur*, or the total leaf area used for the gas exchange measurement from *L. decidua*, *P. sylvestris*, *P. abies* and *V. vitis-idaea*) were subsequently placed into separate envelopes and dried in an oven at 70 °C around 48 h until dry. Fresh weight, dry weight and leaf area were then used to calculate water content and LMA as described by Cornelissen et al. (2003).

### Microscopy and leaf trait analysis

Leaf tissues (mid leaf, avoiding the major veins) from early, mid and late season were prepared for light microscopy (LM) and transmission electron microscopy (TEM). Samples of ~2 × 3 mm were fixed in Karnovskys’s fixative (5% glutaraldehyde, 4% paraformaldehyde and 0.1 M sodium cacodylate buffer) including a vacuum treatment, washed in the buffer and post-fixed in buffered 1% osmium tetroxide. Samples were step-wise dehydrated in a graded acetone series, infiltrated with three different ratios of Spurr resin to acetone and embedded in Spurr resin within flat molds. The resin was polymerized in an oven at 60 °C for 8 h. The samples were sectioned into semi-thin (3 μm) and ultrathin (50 nm) sections for LM and TEM, respectively, using a SuperNova ultra-microtome (Reichert-Jung/LKB) with a diamond knife. Light microscopy sections were left overnight on a hot plate to ensure proper attachment of the section to the glass slide and then stained with either Toluidine Blue, Safranin or periodic acid--Schiff and imaged in a Leica DM5000B microscope. The ultrathin sections were collected on carbon-coated copper grids and contrasted with 1% uranyl acetate and lead citrate (2.7% in 3.5% sodium citrate) and examined in a Philips CM 100 TEM at 80 kV (Philips, Amsterdam, The Netherlands). The LM sections were made for all three time points of the growing season. Only samples from early and mid-season were used for TEM and subsequent cell wall thickness measurements. Hence, the cell wall data were pooled, and TEM data variation was not investigated for seasonal effects.

**Figure 1.**
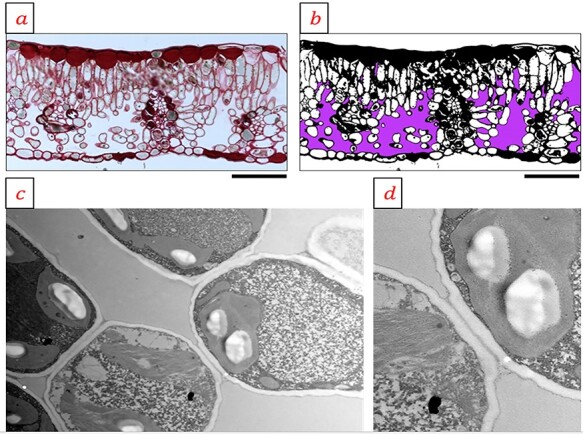
Leaf anatomy and examples of anatomical trait measurements in *B. pendula*. (a) Semi-thin section stained with periodic acid--Schiff and imaged in a brightfield microscope shows the stacked layers of small palisade cells, air-filled spongy mesophyll, the stomata on the lower leaf side and phenol-rich epidermis cells, and (b) after image segmentation for area fraction of intercellular air spaces. (c) Ultra thin section of mesophyll cells imaged in transmission electron microscope visualizing the diffusion path defined by the cells and the intercellular airspaces. (d) Details from an area selected for wall thickness measurements. Bar = 100 μm (a and b), 2 μm (c and d).

Images were analyzed with ImageJ (Schneider et al. 2012) with the ‘Trainable Weka Segmentation’ plugin to measure the area fraction of intercellular airspaces (*F*_ias_) in the mesophyll tissue from LM sections. The plugin allows a manual choice of colors for analysis of the pixel composition of the LM sections (see Figure 1). We used the average of two LM pictures per sample analyzed this way, resulting in a total of three samples per season per species (*n* = 54).

Quantification of mesophyll cell wall thickness (*T*_cw_) was performed from TEM images taken at 7900× magnification (see Figure 1), as done in Nafisi et al. (2015). For each species, 100 measurements were done distributed over three samples (three individual plants) for the mid-season samples, and two to three for the early season samples. As we pooled the data, the *T*_cw_ measurements are from a total of five to six different individuals per species.

### Statistical analysis

Linear regression (ordinary least squares) was used to analyze the effects of *r_m_*, *r_s_* and LMA on *P_r_* as well as the effects of LMA, T_cw_, water content and *F*_ias_ on *r_m_*. Analysis of variance was used to detect differences in *P_r_*, *r_m_*, *r_s_*, *A*_max_, *V*_cmax_, *J*_max_, TPU, *r_d_*, leaf water content, LMA, *F*_ias_ and *T*_cw_ between the species and growing seasons. Where necessary, the data were transformed to meet the assumptions of normality and equal variance, which were tested using Shapiro–Wilk tests and qq-plots. When significant differences between means were detected, Tukey’s honest significant difference test was used to determine which species or growing seasons differed (*P* < 0.05). All calculations were performed using R Studio Version 1.1.456 (RStudioTeam 2015).

## Results

### CO_2_ refixation is highest in evergreens and in climax species

**Figure 2.**
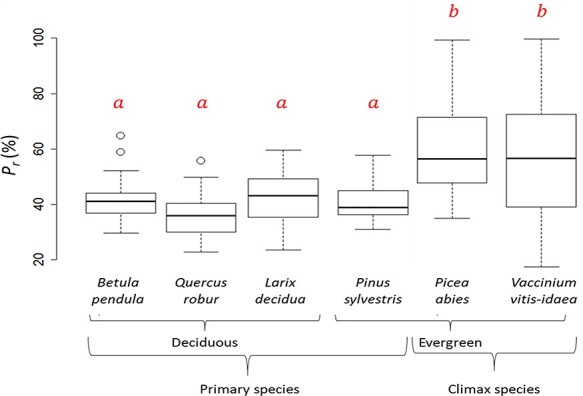
Percentage of CO_2_ refixed (*Pr*) for six different woody species, separated into functional group (deciduous, evergreen, and primary and climax species). The center thick line of each boxplot represents the median. Different letters (a and b) indicate significant statistical difference at the *P* < 0.01 level using Tukey HSD. *N* = 156.

The percentage of (photo)respiratory CO_2_ that was refixed inside the mesophyll (*P_r_*) ranged from about 20 to over 90% (Figure 2). Functional type mattered (Table S1 available as Supplementary data at *Tree Physiology* Online, Figure 2), with the climax species *P. abies* and *V. vitis-idaea* showing significantly greater values of mean *P_r_* (55.5 and 59.6%, respectively) than the early/mid successional species *B. pendula*, *Q. robur*, *L. decidua* and *P. sylvestris*, which displayed mean values of 41.8, 35.7, 42.0 and 40.6% (Figure 2, Table S1 available as Supplementary data at *Tree Physiology* Online). Deciduous species refixed on average significantly less CO_2_, only 40% of the respired CO_2_, while evergreen species on average refixed significantly more CO_2_, about 52% of their respired CO_2_. Deciduous broadleaved species had significantly lower *P_r_* than evergreen conifers, with mean (±SD) values of 38.7 (±8.5)% compared with 50.1 (±15.9)%, (Figure 2). Deciduous conifer species (*L. decidua*) had mean *P_r_* values of 42 (±9.5)%, which was significantly lower than the evergreen broadleaved (*V. vitis-idaea)* values of 55%.

**Figure 3.**
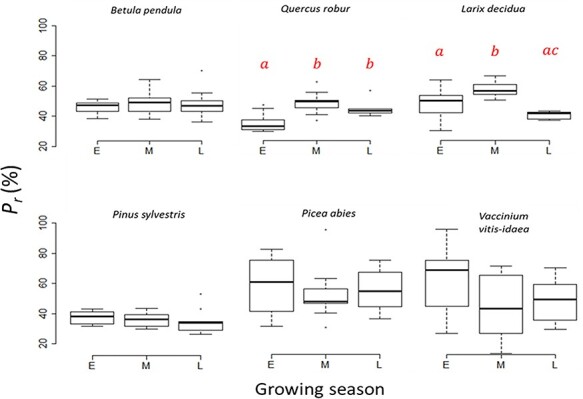
Percentage of CO_2_ refixed (*Pr*) for six different woody species early (E), mid (M) and late (L) in the growing season. The center thick line of each boxplot represents the median. Different letters (a and b) indicate significant statistical difference at the *P* < 0.01 level using Tukey HSD. Differences were tested with Tukey HSD. *N* = 156.

**Figure 4.**
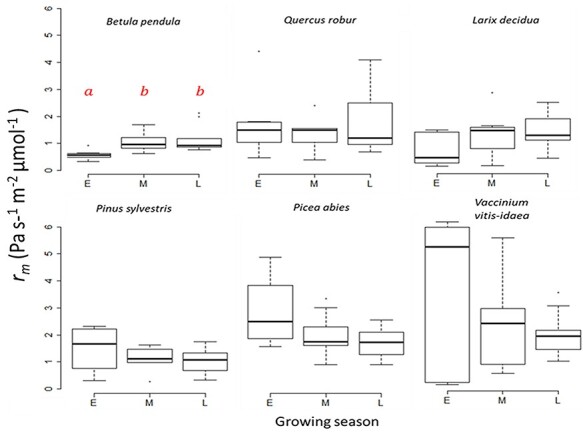
Mesophyll resistance to CO_2_ diffusion (*rm*) of six different woody species early (E), mid (M) and late (L) in the growing season. The center thick line of each boxplot represents the median. Different letters (a and b) indicate significant statistical difference at the *P* < 0.01 level using Tukey HSD. Differences were tested with Tukey HSD. *N* = 136.

We found a highly significant (*P* < 0.01) difference between species for the variables: *P_r_*, *r_m_ r_s_*, *A*_max_, *V*_cmax_, *J*_max_, TPU, *r_d_*, leaf water content, LMA, intercellular airspace and cell wall thickness. In addition, *r_s_*, *A*_max_, *V*_cmax_, *J*_max_, TPU, *r_d_*, leaf water content and LMA were also affected to some degree by seasonality with differences being specifically pronounced between early and late in the season (Table 1, Table S1 available as Supplementary data at *Tree Physiology* Online). The photosynthetic parameters and LMA were lowest early in the growing season, while at the same time, leaf water percentage was highest. Mean values of *P_r_*, however, only changed significantly between seasons for two species: *Q. robur* and *L. decidua* (Figure 3, Table S1 available as Supplementary data at *Tree Physiology* Online). *Quercus robur* was not refixating as much CO_2_ during early season as compared with mid and late season, while *L. decidua* showed the highest percentages in the middle of the season. Although we expected *r_m_* to change throughout the season as the leaves matured, only *B. pendula* had a significantly (*P <* 0.02) lower *r_m_* early in the growing season (Figure 4, Table S1 available as Supplementary data at *Tree Physiology* Online) compared with late season.

**Table 1:** ANOVA results: The effect of species and growing season on leaf traits: *P*_r_ (percentage of respiratory CO_2_ refixed), *r*_m_ (mesophyll resistance to CO_2_ diffusion), *r*_s_ (stomatal resistance to CO_2_ diffusion), *A*_max_ (photosynthetic capacity), *V*_cmax_ (maximum carboxylation rate of Rubisco), *J*_max_ (maximum electron transport rate), TPU (triose phosphate use), *r*_d_ (respiration in the light), leaf water content, LMA (leaf mass per area), *F*_ias_ (average fraction of intercellular airspaces in the mesophyll tissue as estimated from light microscope sections), and *T*_cw_ (average thickness of mesophyll cell wall as measured from transmission electron microscope). F-values and p-values with species and light environment as main effects. Bold numbers represent p-values less than 0.05 (p > 0.05).

Variables	Species		Growing season (early, mid and late)	
	*F*-value	*P*-value	*F*-value	*P*-value
*P_r_* (%)	14.4	**1.6e−11**	0.4	0.7
*r_m_* (p_a_ s^−1^ m^−2^ μmol^−1^)	3.6	**0.0044**	0.9	0.4
*r_s_* (p_a_ s^−1^ m^−2^ μmol^−1^)	15.0	**6.49e−12**	11.9	**1.57e−05**
*A*_max_ (μmol m^−2^ s^−1^)	12.56	**3.5e−10**	5.0	**0.008**
*V*_cmax_ (μmol m^−2^ s^−1^)	20.2	**5.16e−15**	6.0	**0.00324**
*J*_max_ (μmol m^−2^ s^−1^)	13.6	**8.04e−11**	6.5	**0.002**
TPU (μmol m^−2^ s^−1^)	12.5	**4.24e−10**	4.1	**0.018**
*r_d_* (μmol m^−2^ s^−1^)	11.4	**53e−09**	3.8	**0.024**
Leaf water content (%)	7.3	**3.97e−06**	61.2	**<2e−16**
LMA (g m^−2^)	182.1	**<2e−16**	7.8	**6.07e−4**
*T*_cw_ (μm)	28.27	**3.25e−07**	--*	--*
*F*_ias_ (%)	915.7	**4.42–11**	0.3	0.8

^*^Cell wall thickness data is from one point of the growing season (mid-season)

### Cell wall thickness significantly affected *P_r_* and *r_m_*

**Figure 5.**
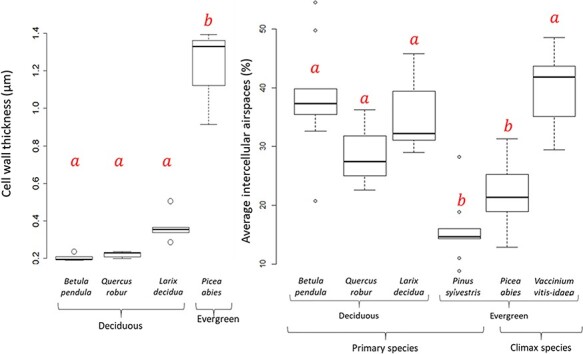
Cell wall thickness (*T*_cw_) of mesophyll cells, and average fraction of intercellular airspace (*F*_ias_) of leaves from different woody species. Which functional group (deciduous, evergreen, and primary and climax species) each species belongs to is indicated. The center thick line of each boxplot represents the median. Different letters (a and b) indicate significant statistical difference at the *P* < 0.01 level using Tukey HSD. *N* = 12 (cell wall thickness), *n* = 54 (average intercellular airspace).

Cell wall thickness (*T*_cw_) was significantly greater in the evergreen *P. abies*, with an average of 1.2 (±0.08) μm, compared with the deciduous species *B. pendula*, *Q. robur* and *L. decidua*, which had a *T*_cw_ of 0.2 (±0.009), 0.22 (±0.02) and 0.39 (±0.08) μm, respectively (Figure 5). Although we had few TEM samples, we observed a significant effect between *T*_cw_ and *P_r_* (*P* = 0.025, *r*^2^ = 0.18) and between *T*_cw_ and *r_m_* (*P* = 0.05, *r*^2^ = 0.15) using an ordinary least squares model. We found no support for a relationship between intercellular airspace percentage in the mesophyll and *P_r_*, or *r_m_* (results not shown), but we observed a species variation that could be explained by functional type (Figure 5, Table 1, Table S1 available as Supplementary data at *Tree Physiology* Online). The coniferous evergreen species had significantly (*P* < 0.01) less air in their mesophyll than broadleaved species and the deciduous conifer (Figure 5). Light microscope pictures showed that average (±SD) intercellular airspace percentage in the mesophyll was about 38.2 (±9.7), 28.8 (±6.9), 35.5 (±6.0), 39.7 (±6.4), 15.8 (±5.5) and 21.7 (±5.1)% for *B. pendula*, *Q. robur*, *L. decidua*, *V. vitis-idaea*, *P. sylvestris* and *P. abies*, respectively (Figures 1 and 5, Table S1 available as Supplementary data at *Tree Physiology* Online).

**Figure 6.**
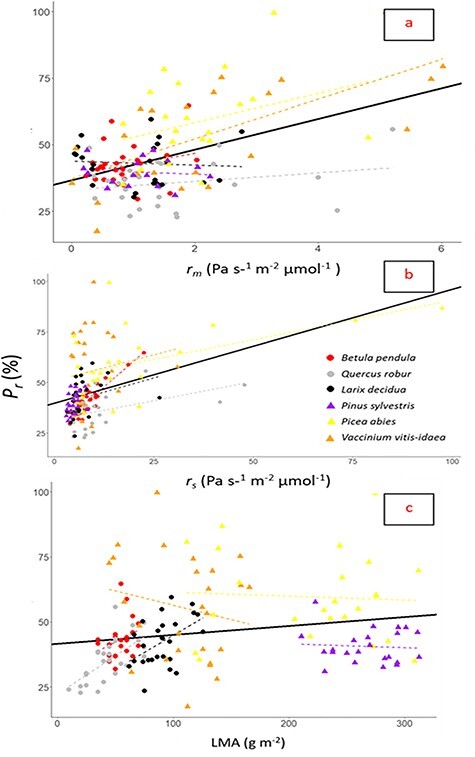
Relationship between the percentages of CO_2_ refixed (*P*_r_) and (a) mesophyll resistance to CO_2_ diffusion (*r*_m_), (b) stomatal resistance to CO_2_ diffusion (*r*_s_) and (c) LMA in six different woody species. Deciduous species are marked with circles, and evergreens are marked with triangles. Regression lines: (a) all species (black solid line, y = 36.7 + 5.8x, *P* < 0.01, adj. *r*^2^ = 0.22), *B. pendula* (dashed red line, y = 38.7 + 4.0x, *P* = 0.22, adj. *r*^2^ = 0.02), *Q. robur* (dashed gray line, y = 33.0 + 1.6x, *P* = 0.25, adj. *r*^2^ = 0.02), *L. decidua* (dashed black line, y = 43.4 + −0.8x, *P* = 0.75, adj. *r*^2^ = −0.04), *P. sylvestris* (dashed purple line, y = 40.6 ± 0.9x, *P* = 0.71, adj. *r*^2^ = −0.06), *P. abies* (dashed yellow line, y = 47.3 + 5.5x, *P* = 0.16, adj. *r*^2^ = 0.05) and *V. vitis-idaea* (dashed orange line, y = 37.3 + 7.5x, *P* < 0.01, adj. *r*^2^ = 0.43); (b) all species (black solid line, y = 39.8 + 0.56x, *P* < 0.01, adj. *r*^2^ = 0.17), *B. pendula* (dashed red line, y = 26.5 + 1.7x, *P* < 0.01, adj. *r*^2^ = 0.784), *Q. robur* (dashed gray line, y = 31.5 + 0.38x, *P* = 0.011, adj. *r*^2^ = 0.19), *L. decidua* (dashed black line, y = 37.6 + 0.62x, *P* = 0.14, adj. *r*^2^ = 0.05), *P. sylvestris* (dashed purple line, y = 32.6 + 1.5x, *P* = 0.06, adj. *r*^2^ = 0.12), *P. abies* (dashed yellow line, y = 51.7 + 0.4x, *P* = 0.01, adj. *r*^2^ = 0.23) and *V. vitis-idaea* (dashed orange line, y = 50.9+ 0.51x, *P* = 0.5, adj. *r*^2^ = −0.02) and (c) all species (black solid line, y = 41.6 + 0.034x, *P* = 0.012, adj. *r*^2^ = 0.035), *B. pendula* (dashed red line, y = 30.7 + 0.23x, *P* = 0.13, adj. *r*^2^ = 0.06), *Q. robur* (dashed gray line, y = 22.28 + 0.29x, *P* < 0.01, adj. *r*^2^ = 0.40), *L. decidua* (dashed black line, y = 19.51 + 0.25x, *P* = 0.025, adj. *r*^2^ = 0.15), *P. sylvestris* (dashed purple line, y = 44.6 – 0.01x, *P* = 0.76, adj. *r*^2^ = −0.04), *P. abies* (dashed yellow line, y = 62.9 – 0.02x, *P* = 0.793, adj. *r*^2^ = −0.04), *V. vitis-idaea* (dashed orange line, y = 67.5 – 0.11x, *P* = 0.344, adj. *r*^2^ = −0.003). *N* = 136.

### *r_m_*, *r_s_* and LMA explain differences in *P_r_*

We found a significant effect on *P_r_* for *r_m_*, *r_s_* and LMA; all had positive correlations (Table 2). Although the relationship between *r_m_* and *P_r_* differed among species, with some species displaying a negative relationship, a highly significant relation was observed across species (Figure 5). This species variation is not surprising considering that multiple morphological and physiological traits affected *P_r_* (Table 2) and that these traits also varied between species and season (Table 1, Table S1 available as Supplementary data at *Tree Physiology* Online).

**Table 2:** Linear regression results: The effect of physiological and morphological traits on the dependent variable, *P*_r_ (percentage of refixed respiratory CO_2_). Model estimate from three (1–3) ordinary least squares linear models, with T-values and p-values in parentheses. Physiological traits: *r*_m_ (mesophyll resistance to CO_2_ diffusion), *r*_s_ (stomatal resistance to CO_2_ diffusion), and morphological trait: LMA (leaf mass per area). Bold numbers represent p-values less than 0.05.

	1	2	3
Independent variables			
*r_m_* (p_a_ s^−1^ m^−2^ μmol^−1^)	5.77 (6.25, **5.13e−09**)	5.04 (5.51, **1.8e−07**)	4.90 (5.61, **1.24e−07**)
*r_s_* (p_a_ s^−1^ m^−2^ μmol^−1^)		0.59 (3.51, **6.2e−04**)	0.63 (3.88, **1.7e−04**)
LMA (g m^−2^)			0.05 (4.2, **4.74e−05**)
Intercept	36.7 (21.0, **<2e−16**)	32.33 (15.7, **<2e−16**)	26.35 (10.5, **<2e−16**)
Degrees of freedom	134	132	126
Adjusted *r*^2^	0.22	0.28	0.36
*F*-statistic	39.04	27.55	25.59
*P*-value	**5.135e−9**	**1.005e−10**	**5.375e−13**

## Discussion

### Evergreen and climax species are efficient in using their respiratory CO_2_

We showed that the two evergreen species *P. abies* and *V. vitis-idaea* had the highest *P_r_* of all species measured (Figure 2). Efficient refixation of (photo)respiratory CO_2_ will be an advantage when stomata are closed, such as during drought, high temperature stress or during winter-hibernating periods. Conifers growing in mild winter climates can have significant carbon fixation all year round (Fry and Phillips 1977, Harrington et al. 1994), while conifers in colder, northern environments seem to limit carbon fixation to irregular periods with temperatures above freezing (Jurik et al. 1988, Schaberg et al. 1995). Being able to utilize even a small amount of CO_2_ without having to open stomata might be an advantage for evergreens, especially since there are examples of water stress (Kincaid and Lyons 1981, Teskey et al. 1984) and stomatal closure (Delucia 1987) being limiting factors of winter photosynthesis.

Although also an evergreen, *P. sylvestris* had refixation percentages that were more similar to the deciduous species. *Picea abies* and *V. vitis-idaea* can be defined as climax species, or ‘late’ successional species, while *P. sylvestris* and the deciduous species are all pioneer, or ‘early’ successional species in Sweden (Hannon et al. 2018). It is therefore possible, and a potential subject for further investigation, that climax species overall have higher *P_r_* compared with the early successional species. It has been shown that early and late successional species can have different physiological capabilities; for example, early successional species seem to have higher net photosynthetic rates, both on an area and mass basis (Kloeppel et al. 1993, Matsuki and Koike 2006, Bussotti 2008). *P_r_* could on the other hand be a trait that is more prominent in late succession.

### Refixation percentage is positively correlated with *r_m_*, *r_s_* and LMA

A central point of our results was that higher *r_m_*, *r_s_* and LMA correlated with higher *P_r_* (Figure 5). There were large variations in the data when all species were pooled, with some species even displaying a negative relationship to one or more of the above traits. However, all species that had significant relationships between *P_r_* and *r_m_*, *r_s_* and LMA had positive correlations similar to the average trend (Figure 6). It is possible that some of the same adaptations or physiological states that increase *r_m_* and LMA also increase *P_r_*. Alternatively, *r_m_* and LMA directly affect *P_r_*. Both high *r_m_* and high *r_s_* can decrease photosynthetic activity due to a slowdown of CO_2_ diffusion to the photosynthetic sites from the exterior (Parkhurst 1994, Warren 2008, Jimei et al. 2017). This is the basis for our underlying hypothesis: a long, low-conductive pathway for CO_2_ diffusion (high *r_m_*) from the cell to the atmosphere, and closed stomata (high *r_s_*), would help capture (photo)respiratory CO_2_ produced in the mesophyll, increasing *P_r_*. Our results are in line with this hypothesis. However, they also suggest that more factors influence *P_r_*, which should be further expanded on in later studies. The same methods have previously been applied on some of the same species studied here (Eckert et al. 2020). There was no significant correlation between *P_r_* and *r_m_* in the findings of Eckert et al. (2020); however, there was a positive trend. The previous article did find a correlation between *V*_cmax_ that was not obvious in the current study. It is possible that more data are needed in order to make larger-scale correlations obvious.

It should be mentioned that the LeafWeb model (see Materials and methods) is using both stomatal and leaf conductance to calculate *P_r_*. These factors are thus not independent from each other. The model assumes that changes in *r_m_* and *r_s_* lead to changes in *P_r_*. While experimental validation is needed, the assumptions are justifiable because carboxylation does not differentiate between CO_2_ sources, whether from mitochondria or intercellular airspace. What should matter to the fate of mitochondria-derived CO_2_ is the relative diffusion-resistance to the carboxylation sites versus relative resistance to the outside of the leaf. In this regard, the fact that LMA (and cell wall thickness) had a positive correlation with *P_r_* gives some evidence that this assumption is correct. Furthermore, the study by Busch et al. (2013) measured refixation directly and found that a continuous layer of chloroplasts covered the cell periphery. The chloroplasts captured the (photo)respired CO_2_ and boosted photosynthesis. Similarly, high efflux resistance due to chloroplast positioning is one of the assumptions made by the LeafWeb model. Busch et al. (2013) also found that refixating (photo)respiratory CO_2_ boosted photosynthesis more when ambient atmospheric CO_2_ concentrations were low (200 μmol^−1^). Efficiently recycling mitochondria-derived CO_2_ has implications for water-use efficiency. During drought, high *P_r_* would allow species to close stomata while keeping up a limited amount of photosynthesis. The meta-study by Ainsworth and Long (2005) showed that plants overall increase *r_s_* when exposed to elevated CO_2_. This might make (photo)respiratory-derived CO_2_ a larger fraction of the CO_2_ used in photosynthesis, especially in high *r_m_* species, such as the evergreens in this study.

We expected LMA to correlate with *P_r_* because it can be a predictor of thick cell walls and tightly packed mesophyll (Niinemets et al. 2009, Onoda et al. 2017), which simultaneously enhances *r_m_* (Flexas et al. 2008). Although LMA also increases with more mesophyll layers, this does not necessarily increase *r_m_* if *r_m_* is measured per unit of leaf area (supplementary material in Sun et al. 2014*b*). In our data, *r_m_* and LMA were not correlated, which might be due to *r_m_* being measured on a leaf-area basis. A new paper by Veromann-Jürgenson et al. (2020) found that variation in LMA did not correlate to structural traits known to control *r_m_* across species. Veromann-Jürgenson et al. (2020) concluded that more detailed knowledge of the underlying traits affecting *r_m_* are needed for accurate prediction, and further showed evidence that chloroplast area exposed to intercellular airspaces and cell wall thickness are important drivers of *r_m_*. Likely, these two traits are therefore also important drivers of *P_r_*.

Environmental factors should also be considered, as they have been shown to rapidly induce changes in *r_m_* (but not in LMA) for different species: increases in temperature generally lower *r_m_* until a certain threshold (Bernacchi et al. 2002), and soil water availability, salinity and growth irradiance may all affect *r_m_* (Flexas et al. 2008, Flexas et al. 2009). It would therefore be interesting to further investigate these elements in relation to *P_r_* to further clarify if *r_m_* is directly affecting *P_r_*, or another factor affecting both *P_r_* and *r_m_* simultaneously.

### Season, cell wall thickness and fraction of intercellular airspace

The point in the growing season (early, mid or late) at which the measurements were taken did not seem to matter for *r_m_* and was only significant for *P_r_* in *L. decidua* and *Q. robur*, both of which showed highest refixation mid-season (Figure 3). However, most of the other physiological and morphological traits investigated did change throughout the growing seasons (*r_s_*, *A*_max_, *V*_cmax_, *J*_max_, TPU, *r_d_*, leaf water content and LMA, Table 1, Table S1 available as Supplementary data at *Tree Physiology* Online). This is in line with previous findings that mature leaves from both mid and late successional species had higher photosynthetic rates than young leaves (Yu et al. 2020). The fact that so many other traits did change throughout the growing season shows that the leaves underwent some maturation, but that it did not have a great effect on *P_r_* and *r_m_*. On the other hand, both *L. decidua* and *Q. robur* refixed more of their (photo)respiratory CO_2_ in the middle of the growing season, which means that for these two species, leaf maturation might have had an effect. In addition to anatomical and physiological traits, it is likely that the environmental conditions around leaves influence *P_r_*. Particularly temperature and irradiance, as they are known to affect both respiration and photosynthesis, and as they have been shown to strongly correlate with refixation in photosynthetic bark of *Pinus monticola* (Cernusak and Marshall 2001). Another explanation for a seasonal effect on *P_r_* for *L. decidua* and *Q. robur* might therefore be that these two species had a different response to the mid-season climate than the others.

Even though we do not have as many TEM pictures as *P_r_* measurements, we did find a correlation between cell wall thickness and *r_m_*. Of the anatomical traits investigated in our study, cell wall thickness is known to have a strong positive correlation with *r_m_* (Tomás et al. 2013, Onoda et al. 2017, Veromann-Jürgenson et al. 2017). Species differences were found where *P. abies* had the thickest cell walls (Figure 5). It is possible that a stronger correlation would have emerged if species and growing season samples had been represented. This is also true for cell wall thickness and *P_r_*, where we also found a significant, positive effect. *Picea abies* and *V. vitis-idaea* were the two species with the highest *P_r_*. While we have no data on cell wall thickness for *V. vitis-idaea*, *P. abies* clearly also has thicker cell walls than the other species tested (Figure 5). In the literature, a close relative of *V. vitis-idaea*, *Vaccinium oxycoccus*, shows a cell wall thickness of 0.61–1.06 μm. If similar numbers can be found for *V. vitis-idaea*, it would be close to *P. abies* and significantly thicker than the deciduous species (Figure 5). Thick cell walls can thus be a predictor of high *P_r_*. More data on cell wall thickness for different species would help investigate this further.

Comparing intercellular airspace between species shows that the two evergreen conifers had much more tightly packed mesophyll than the others, which is in line with expectations given their sturdy leaf anatomy (Niinemets et al. 2009). Intercellular airspace percentage has in some cases been shown to have an influence on *r_m_*. However, it seems largely to be determined as only affecting *r_m_* to a small degree, as CO_2_ diffusion in air does not meet much resistance compared with diffusion across cell walls (Tholen et al. 2012). This might then be the same reason it did not affect *P_r_*.

## Conclusions

When studying the percentage of respiratory CO_2_ refixation (*P_r_*) between different species and functional types, we found that evergreen, late successional species, especially *V. vitis-idaea* and *P. abies*, utilized significantly more of their mitochondria-derived CO_2_ than deciduous and early successional species. Measuring *P_r_* at various points of the growing season showed that *P_r_* was relatively constant and that the two evergreen species had the highest percentages of *P_r_* throughout the entire season. Among the anatomical and physiological traits we investigated, mesophyll resistance to CO_2_ diffusion (*r_m_*), stomatal resistance to CO_2_ diffusion (*r_s_*) and leaf dry matter content (LMA) were significantly, positively correlated with *P_r_*. We suggest that this is due to higher *r_m_* and *r_s_* decreasing diffusion of (photo)respiratory CO_2_ out of the leaf. Cell wall thickness was significantly different between conifers and broadleaves, and cell wall thickness positively affected both *P_r_* and *r_m_* in our study.

Our findings suggest that species with higher *r_m_* and thicker cell walls might be more efficient with their mitochondria-derived CO_2_. *P_r_* should be considered when modeling the overall CO_2_ fertilization effect for terrestrial ecosystems dominated by high-*r_m_* species. This is because some species develop a lower number of stomata under prolonged elevated atmospheric CO_2_, or close more of the stomata (increasing *r_s_*). They might in that way be able to keep photosynthetic rates high enough while also preserving water. There are thus implications for water-use efficiency and overall CO_2_ drawdown.

## Supplementary Material

Supplementary_information_tpab016
