# Supplementary material for: CO2 refixation is higher in leaves of woody species with high mesophyll and stomatal resistances to CO2 diffusion
Source: Tree Physiol. 2021 Feb 17;41(8):1450–61. doi: 10.1093/treephys/tpab016 (PMC8359682; doi:10.1093/treephys/tpab016)
Supplement: Supplementary_information_tpab016 [file supplementary_information_tpab016.docx]

**Supplementary information**

Table S1. Average measures of different leaf traits early, mid, and late in the growing season for different woody species: *P_r_* (percentage of respiratory CO_2_ refixed), *r_m_* (mesophyll resistance to CO_2_ diffusion), *r_s_* (stomatal resistance to CO_2_ diffusion), *A_max_* (maximum photosynthesis rate), *V_cmax_* (maximum rate of carboxylation of Rubisco), *J_max_* (the maximum rate of electron transport), TPU (triose phosphate utilization), LMA (leaf mass per area), water content (ratio of fresh weight and oven-dried weight), F_ias_ (average percentage of intercellular airspaces in the mesophyll tissue as estimated from light microscope sections), and T_cw_ (average thickness of mesophyll cell wall as measured from transmission electron microscope). Different letters (a, b and c) indicate significant statistical difference between seasons where it was found, at the *p* < 0.05 level using Tukey HSD. Averages are given ±SD, n = 156.

| Variables | Season | | | |
| --- | --- | --- | --- | --- |
|  | **Early** | **Mid** | **Late** | **Species mean** |
| *P_r_* (%)  *B. pendula*  *Q. robur*  *L. decidua*  *P. sylvestris*  *P. abies*  *V. vitis-idaea*  Season means | 39.9 ± 3.9  28.6 ± 6.2 a  41.7 ± 9.9 a  42.4 ± 4.5  62.4 ± 20.8  64.3 ± 22.8  45.6 ± 18.1 | 43.1 ± 8.5  41.8 ± 7.6 b  50.7 ± 5.0 b  40.6 ± 5.0  57.3 ± 18.2  48.5 ± 21.8  46.9 ± 13.4 | 42.5 ± 10.1  37.5 ± 4.8 b  33.7 ± 2.5 c  39.4 ± 8.5  59.7 ± 13.9  52.8 ± 14.1  44.2 ± 13.0 | 41.8 ± 7.7  35.7 ± 8.3  42.0 ± 9.5  40.6 ± 6.3  60.0 ± 17.1  55.5 ± 20.2 |
| *r_m_* (p_a_ s^-1^m^-2^µmol^-1^)  *B. pendula*  *Q. robur*  *L. decidua*  *P. sylvestris*  *P. abies*  *V. vitis-idaea*  Season means | 0.47 ± 0.17 a  1.48 ± 1.17  0.68 ± 0.62  1.42 ± 0.91  2.79 ± 1.44  3.40 ± 3.09  1.5 ± 1.6 a | 0.99 ± 0.38 b  1.65 ± 1.45  1.24 ± 0.86  1.02 ± 0.53  1.98 ± 0.76  2.18 ± 1.66  1.5 ± 1.1 a | 1.09 ± 0.52 b  1.63 ± 1.15  1.34 ± 0.66  0.95 ± 0.49  1.64 ± 0.55  1.84 ± 0.84  1.4 ± 0.8 a | 0.85 ± 0.46  1.59 ± 1.21  1.10 ± 0.75  1.09 ± 0.61  2.00 ± 0.91  2.32 ± 1.84 |
| *r_s_* (P_a_s^-1^ m^-2^ µmol^-1^)  *B. pendula*  *Q. robur*  *L. decidua*  *P. sylvestris*  *P. abies*  *V. vitis-idaea*  Season means | 8.7 ± 1.5  11.7 ± 10.8  8.4 ± 6.5  4.9 ± 1.3  43.2 ± 31.3 a  12.2 ± 7.5  14.0 ± 17.1 a | 9.1 ± 4.2  14.4 ± 13.7  8.0 ± 3.2  5.5 ± 1.3  12.2 ± 4.5 b  7.9 ± 2.9  9.6 ± 6.9 ab | 8.9 ± 5.8  7.0 ± 2.4  4.8 ± 1.0  4.3 ± 1.5  9.0 ± 4.8 b  6.8 ± 3.9  6.9 ± 4.0 b | 8.9 ± 4.0  11.1 ± 10.3  7.2 ± 4.6  4.9 ± 1.4  20.2 ± 22.3  9.1 ± 5.6 |
| *A_max_* (µmol m^-2^ s^-1^)  *B. pendula*  *Q. robur*  *L. decidua*  *P. sylvestris*  *P. abies*  *V. vitis-idaea*  Season means | 17.2 ± 2.7 a  9.9 ± 2.3 a  25.5 ± 5.7  33.7 ± 8.6  8.6 ± 1.8 a  16.6 ± 6.4  18.0 ± 9.3 a | 18.5 ± 2.7 b  14.7 ± 3.0 ab  27.6 ± 8.3  26.7 ± 14.4  21.9 ± 4.6 b  16.0 ± 6.8  20.9 ± 8.9 b | 21.5 ± 3.1 b  18.3 ± 4.6 b  19.3 ± 6.7  39.2 ± 25.8  27.2 ± 3.2 c  21.1 ± 8.3  24.5 ± 13.5 b | 19.0 ± 3.3  14.2 ± 4.8  24.2 ± 7.5  33.1 ± 18.7  19.8 ± 8.4  18.0 ± 7.3 |
| *V_cmax_* (µmol m^-2^ s^-1^)  *B. pendula*  *Q. robur*  *L. decidua*  *P. sylvestris*  *P. abies*  *V. vitis-idaea*  Season means | 31.1 ± 4.2 a  17.2 ± 5.9 a  39.0 ± 12.7  59.2 ± 11.8  9.7 ± 5.3 a  15.1 ± 5.8 a  26.8 ± 9.3 a | 36.0 ± 5.4 ab  27.2 ± 7.8 b  52.5 ± 12.8  47.3 ± 13.5  31.0 ± 11.9 b  17.5 ± 8.6 ab  20.9 ± 8.9 b | 39.9 ± 9.1 b  36.1 ± 9.3 c  45.0 ± 12.3  46.8 ± 19.1  33.0 ± 13.0 b  27.4 ± 11.6 b  24.5 ± 13.5 b | 35.5 ± 7.3  26.5 ± 10.9  44.0 ± 13.0  50.2 ± 15.6  26.2 ± 14.6  20.2 ± 10.3 |
| *J_max_* (µmol m^-2^ s^-1^)  *B. pendula*  *Q. robur*  *L. decidua*  *P. sylvestris*  *P. abies*  *V. vitis-idaea*  Season means | 33.2 ± 4.4  17.9 ± 5.1 a  49.5 ± 8.3  64.4 ± 16.2  12.6 ± 5.5 a  23.9 ± 3.9 a  18.7 ± 5.9 a | 34.9 ± 6.0  26.8 ± 7.7 ab  49.0 ± 15.8  48.3 ± 22.1  33.5 ± 6.0 b  25.1 ± 9.7 ab  36.3 ± 15.4 ab | 40.6 ± 10.4  35.6 ± 10.2 b  40.3 ± 14.5  54.9 ± 17.9  51.4 ± 14.4 c  40.4 ± 17.8 b  43.4 ± 15.3 b | 36.2 ± 7.8  26.5 ± 10.6  46.0 ± 13.4  54.8 ± 19.5  32.5 ± 18.6  30.5 ± 14.2 |
| TPU (µmol m^-2^ s^-1^)  *B. pendula*  *Q. robur*  *L. decidua*  *P. sylvestris*  *P. abies*  *V. vitis-idaea*  Season means | 5.6 ± 0.9 a  3.0 ± 0.7 a  8.6 ± 2.2  11.5 ± 3.4  2.8 ± 0.6 a  5.4 ± 2.2  5.9 ± 3.3 a | 6.0 ± 1.0 ab  4.7 ± 1.1 b  9.1 ± 3.3  6.3 ± 2.6  7.0 ± 1.9 b  5.4 ± 2.5  6.8 ± 3.2 a b | 7.1 ± 1.3 b  5.9 ± 1.7 b  8.9 ± 5.2  11.1 ± 5.8  9.2 ± 1.9 c  7.1 ± 3.1  7.7 ± 3.4 b | 6.2 ± 1.2  4.5 ± 1.7  8.1 ± 2.9  10.4 ± 5.0  6.5 ± 3.0  6.0 ± 2.7 |
| LMA (g m^-2^)  *B. pendula*  *Q. robur*  *L. decidua*  *P. sylvestris*  *P. abies*  *V. vitis-idaea*  Season means | 45.5 ± 7.6 a  28.0 ± 10.6 a  88.3 ± 18.9  277.1 ± 45.1  132.4 ± 15.6 a  67.7 ± 19.0 a  89.0 ± 73.1 a | 57.1 ± 8.6 b  55.6 ± 12.4 b  99.1 ± 17.2  270.6 ± 18.7  252.3 ± 31.5 b  127.9 ± 16.5 b  148.45 ± 91.1 b | 60.7 ± 7.9 b  55.6 ± 15.7 b  88.6 ± 13.8  259.3 ± 29.3  266.0 ± 27.0 b  133.8 ± 19.1 b  146.0 ± 90.5 b | 53.3 ± 10.3  45.7 ± 18.3  91.7 ± 17.0  267.6 ± 29.3  221.9 ± 64.1  109.1 ± 35.5 |
| Water content (%)  *B. pendula*  *Q. robur*  *L. decidua*  *P. sylvestris*  *P. abies*  *V. vitis-idaea*  Season means | 65.2 ± 3.4 a  75.1 ± 9.0 a  72.3 ± 6.0 a  52.5 ± 3.0 a  78.8 ± 2.2 a  77.9 ± 2.1 a  71.0 ± 9.6 a | 59.6 ± 3.7 b  59.6 ± 3.4 b  62.0 ± 3.6 b  51.8 ± 3.8 a  59.9 ± 1.8 b  57.7 ± 2.5 b  58.3 ± 4.5 b | 59.3 ± 4.1 b  56.5 ± 3.4 b  64.0 ± 5.3 b  57.4 ± 2.9 b  58.4 ± 2.1 b  55.0 ± 2.7 b  58.3 ± 4.4 b | 61.9 ± 4.6  64.1 ± 10.2  66.6 ± 6.8  54.1 ± 4.1  64.9 ± 9.3  63.8 ± 10.8 |
| T_cw_ (µm)*  *B. pendula*  *Q. robur*  *L. decidua*  *P. sylvestris*  *P. abies*  *V. vitis-idaea*  Season means | N/A  N/A  N/A  N/A  N/A  N/A  N/A | N/A  N/A  N/A  N/A  N/A  N/A  N/A | N/A  N/A  N/A  N/A  N/A  N/A  N/A | 0.201 ± 0.009  0.216 ± 0.019  0.388 ± 0.081  N/A  1.212 ± 0.261  N/A |
| F_ias_ (%)  *B. pendula*  *Q. robur*  *L. decidua*  *P. sylvestris*  *P. abies*  *V. vitis-idaea*  Season means | 35.9 ± 14.6  28.8 ± 6.9  37.7 ± 7.7  17.9 ± 9.1  25.0 ± 5.8  43.3 ± 1.2  30.8 ± 11.0 a | 42.3 ± 11.2  N/A  30.7 ± 1.6  14.1 ± 5.0  17.4 ± 4.0  39.2 ± 9.5  28.0 ± 13.1 a | 36.4 ± 1.1  N/A  38.2 ± 5.5  15.5 ± 0.7  22.0 ± 2.8  37.4 ± 6.3  29.4 ± 10.0 a | 38.2 ± 9.7  28.8 ± 6.9  35.5 ± 6.0  15.8 ± 5.5  21.7 ± 5.1  39.7 ± 6.4 |

*****Cell wall thickness data is from one point of the growing season (mid-season)
